# Isolation of keratinophilic fungi from the soil of Greater Tunb, Abu-Musa, and Sirri islands in the Persian Gulf, Iran

**DOI:** 10.18869/acadpub.cmm.3.2.13

**Published:** 2017-06

**Authors:** M Nosratabadi, P Kordbacheh, R Kachuei, M Safara, S Rezaie, MA4 Afshari

**Affiliations:** 1 Department of Medical Parasitology and Mycology, School of Public Health, Tehran University of Medical Sciences, Tehran, Iran; 2 Molecular Biology Research Center, Baqiyatallah University of Medical Sciences, Tehran, Iran; 3 Division of Molecular Biology, Department of Medical Mycology and Parasitology, School of Public Health, Tehran University of Medical Sciences, Tehran, Iran; 4 Arya Tina Gene Biopharmaceutical Company, Tehran, Iran

**Keywords:** Abu Musa, Greater Tunb, Keratinophilic fungi, PCR, Sirri, Soil

## Abstract

**Background and Purpose:**

Keratinophilic fungi are among the important groups of fungi living in the soil. This study aimed to isolate and identify keratinophilic fungi from the soil of three Iranian islands, namely Greater Tunb, Abu Musa, and Sirri, located in the Persian Gulf using morphological and molecular (polymerase chain reaction) methods.

**Materials and Methods:**

In this study, a total of 60 soil samples were collected from the three islands of Greater Tunb, Abu Musa, and Sirri. The samples were analyzed for the presence of the keratinophilic fungi using a hair baiting technique. Furthermore, the identification of keratinophilic fungi was accomplished through the employment of molecular and sequencing techniques.

**Results:**

A total of 130 fungal isolates, including 11 genera with 24 species, were collected. Accordingly, *Chrysosporium tropicum* (24;18.5%), *C. keratinophilum* (17; 13.1%), *Chrysosporium* species (15; 11.5%), *Aspergillus * species ( 8;6.1%), *Aspergillus flavus* (8; 6.1%), *Penicillium * species (8;6.1%), *Alternaria* spp ( 6; 4.6%), *Phoma * species (5; 3.8%), *Aphanoascus verrucosus* (4;3.1%),* Fusarium chlamydosporum *(4; 3.1%), *Aspergillus trreus *(4;3.1%), *Acremonium * species (4; 3.1%), and other fungi( 23; 17.8 %) isolates were identified . All isolates of keratinophilic fungi were isolated from the soils with the pH range of 7-9.

**Conclusion:**

The results of this study contributed towards a better conceptualization of the incidence pattern of keratinophilic fungi in the regions of Iran. Given that no study has investigated this issue, the findings of the present study can be beneficial for the management of public health surveillance, physicians, and epidemiologists.

## Introduction

Fungi are a group of microorganisms with a wide distribution in soil. These organisms play an important role in the soil ecosystem and soil-borne fungal diseases [1]. A number of soil fungi are known as potential pathogens for humans and animals [2]. Keratinophilic fungi are among important groups of fungi living in the soil that colonize in various keratinous substrates, produce keratinases, and decompose them into components with lower molecular weight [3, 4]. 

The ability of these microorganisms to invade and colonize on the keratinous tissues is closely associated with their ability to use keratin [5]. Dermatophytes are a group of keratinophilic fungi that are often anthropophilic or zoophilic in their natural habitat. However, some of these fungi occur in the soil as saprophytes, which are termed as geophilic dermatophytes [6]. On the other hand, the non-dermatophyte fungi, such as *Aspergillus flavus*, *Fusarium Oxysporum,* and *Chrysosporium* species have the ability to colonize around hair and are isolated from the cutaneous lesions of humans and animals as opportunistic agents [7, 8]. 

Keratinophilic fungi have a variable distribution in the environment depending on such natural factors as keratin sources, soil pH, temperature, humidity, environmental light, and climate [9, 10]. Vanbreu-seghem was the first one detecting the existence of keratinophilic fungi in the soil in 1952 [11]. In the recent years, many studies have been carried out in different parts of the world to study the distribution of these fungi in soil [12-14]. Accordingly, several studies have been conducted in Iran targeting this domain[15-17]. However, there are no data regarding keratinophilic fungi in the soil of Greater Tunb, Abu Musa, and Sirri islands. 

In this study, the identification and chara-cterization of the isolated keratinophilic species were performed using the molecular techniques along with traditional methods. To this aim, the internal transcribed spacer (ITS) region of ribosomal DNA was amplified and the polymerase chain reaction (PCR) products were sequenced. This region is the most widely sequenced DNA region in the molecular ecology of fungi and has been recommended as the universal fungal barcode sequence. It has typically been most useful for molecular systematics at the species level and even within species due to its higher degree of variation than that of other genic regions of rDNA [18]. 

With this background in mind, the present study was carried out with the aim of isolating keratinophilic fungi from the soil of the Iranian islands of Greater Tunb, Abu Musa, and Sirri. The findings of this study can be helpful since no study has investigated this issue in the given regions. 

## Materials and Methods

***Geographical characteristics of the studied islands***

Greater Tunb, Abu Musa, and Sirri islands are located at the Persian Gulf in the most southern part of Iran. These three islands are considered as part of Hormozgan province. The Greater Tunb (10.3 km^2^ wide) has a longitude and latitude of 55˚ 28-55˚ 34 and 26˚ 34-26˚ 30 respectively. Abu Musa Island (12 km^2^ wide) has a longitude and latitude of 54˚ 26-55˚ 19 and 25˚ 51-26˚ 19, respectively. Furthermore, Sirri Island is situated 76 km from Bandar-e Lengeh and 50 km west of Abu Musa Island. This island is almost 5.6 km long with a width of about 3 km. It covers an area of17.3 km². All three islands have a warm and humid climate [19].

***Sample collection***

This descriptive study was conducted in the second half of 2011 in three Iranian islands of Greater Tunb, Abu-Musa, and Sirri. A total of 60 soil samples (i.e., 20 samples from each island) were collected. The samples were collected from the superficial layer of soil with the maximum depth of 10 cm and weight of 300-500 g. During the sampling, necessary accuracy was considered to provide samples from different locations and from places not directly exposed to sunlight. The samples were placed in sterile polyethylene bags, transported to the laboratory, and stored at low temperature (4°C) until tested. The pH of soil samples was measured immediately in a 1:5 soil/deionized water suspension (w/v) using a pH meter. 

***Isolation and identification of isolated fungi***

In this study, the isolation of keratinophilic fungi from soil was performed using the hair baiting technique [11]. After mixing soil samples, 70-100 g of soil was transferred to sterile large deep glass plates. Subsequently, 1 g of a mixture of sterile human child girls’ hairs and horse was added and distributed evenly on the whole surface of the soil. Then, almost 20 cc of sterile distilled water was added and kept for 6-8 weeks at ambient temperature (i.e., 25°C). 

The isolation was carried out by direct transfer of mycelium from the baits to sabouraud dextrose agar medium (Merck, Germany) with chloramphenicol (0.1 mg/mL; Sigma-Aldrich, USA) as well as sabouraud dextrose agar medium with cycloheximide (1 mg/mL; Sigma-Aldrich, USA) and chloramphenicol (0.1 mg/mL). They were then incubated at room temperature for a period of two weeks. The identification of keratinophilic fungi was carried out according to standard procedures [20, 21]. In addition, the unknown fungal isolates were identified through performing DNA sequence analysis.

***Molecular identification***

***DNA extraction***

DNA was extracted following Lee *et al. *[22] with slight modifications. To this aim, the harvested mycelial mass was flash-frozen in liquid nitrogen and ground to a fine powder in a porcelain mortar. The mycelial powder was suspended in DNA extraction buffer containing 1 mM NaCl, 10 mM Tris (pH 8.0), 1 mM ethylenediaminetetraacetic acid, 1% SDS, and 1% Triton X-l00, and then the DNA was extracted. The quality and quantity of the extracted DNA were evaluated using electrophoresis and Nano- Drop, respectively.

***Polymerase chain reaction***

The ITS1-5.8S-ITS2 rDNA was amplified using ITS1 and ITS4 as forward and reverse primers following White *et al.* [23]. The amplification was performed in a total volume of 25 μL in each tube containing 12.5 µL master mix (Ampliqon, Denmark) (buffer, dNTP, Taq DNA polymerase, 2 mM MgCl_2_), 0.5 μL of the template DNA, 1.5 µL of each primer (Cinaclone, Iran) ITS1, namely 5'-TCCGTAGGTGAACCTGCGG-3' and ITS4: 5'-TCCTCCGCTTATTGATATGC-3 '(20 pmol final concentration of each primer), and 9 𝜇L distilled water. 

**Figure 1:**
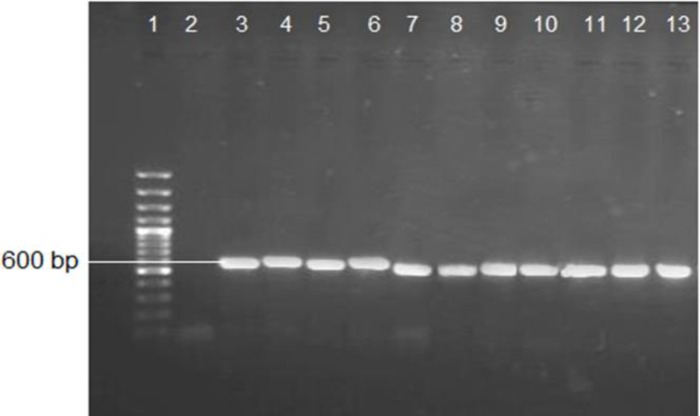
Agarose gel electrophoresis and polymerase chain reaction products of many unknown keratinophilic fungi, lane 1: 100 bp DNA ladder, lane 2: negative control, lane 3: positive control (*Aspergillus flavus*), lane 4: *Aphanoascus verrucosus, *lane 5: *Chaetomium*sp, lane 6: *Phoma*sp, lane 7:* A. terreus*, lane 8: *Alternaria alternate*, lane 9:* Chrysosporiumindicum*, lane 10: *C. keratinophilum,* lane 11: *C. keratinophilum*, lane 12: *C. tropicum*, lane 13: *C. tropicum*

The PCR reaction was carried out using a thermal cycler (Biometra, Germany) with initial denaturation at 94°C for 5 min, 35 cycles with denaturation at 94°C for 30 sec, annealiation at 56°C for 45 sec, extension at 72°C for 45 sec, and a final extension step of 7 min at 72°C. The amplified products were visualized by electrophoresis in 1% agarose gels (CONDA, Spain) using the SYBR Safe stain (Figure 1). The PCR products were sent for sequencing (Macrogen, South Korea) in both directions. The sequences were aligned using Mega 6, followedby visual inspection and manual adjustment. Subsequently, the data were compared with those in the NCBI/GenBank database.

## Results

Out of the 80 soil samples screened for the presence of dermatophytes and keratinophilic fungi, 37 (61.6%) samples were positive for fungal growth (Table 1). A total of 130 fungal isolates, including 11 generawith 24 species, were isolated. They included 24 (18.5%)* Chrysosporium tropicum*, 17 (13.1%) *C. keratinophilum*, 15 (11.5%) *Chrysosporium* species, 8 (6.1%) *Aspergillus* species, 8 (6.1%) *A. flavus*, 8 (6.1%) *Penicillium* species, 6 (4.6%) *Alternaria* species,5 (3.8%) *Phoma* species, 4 (3.1%) *Aphanoascus verrucosus,* 4 (3.1%) *Fusarium chlamydosporum*, 4 (3.1%) *A. trreus*, 4 (3.1%) *Acremonium* species, and 23 (17.8 %) other fungi.

In our study, the majority of keratinophilic fungi (39.2%) were isolated from Greater Tunb Island. Furthermore, most of *Chrysosporium* species (41.4%) were isolated from Abu Musa Island. The isolated fungal species and their incidence rates in each of the studied islands are illustrated in Table 1. The pH of all the keratinophilic fungi isolated from the soils was within 7-9. More details about the isolates and soil pH are presented in tables 2-4.

**Table 1:** Distribution of keratinophilic fungi isolated from the soil samples of Iranian islands of Greater Tunb, Abu Musa, and Sirri islands by mycological and molecular methods

	**Islands**						**Total**	**% Frequency**
	**Greater Tunb**		**Abu Musa**		**Sirri**			
**No. of samples examined**	20		20		20		60	
**No. of positive samples**	15		13		9		37	
**Percentage of positive samples**	75		65		45		61.6	
**Species isolated**	**n**	**%**	**n**	**%**	**n**	**%**	**n**	**%**
*Acremonium* species	2	3.9	2	4.4	0	0	4	3.1
*A. alternata*	2	3.9	0	0	0	0	2	1.5
*Alternaria * species	4	7.8	0	0	2	5.9	6	4.6
*Aphanoascus verrucosus*	4	7.8	0	0	0	0	4	3.1
*A. flavus*	5	9.8	0	0	3	8.8	8	6.1
*A. fumigatus*	3	5.9	0	0	0	0	3	2.3
*A. niger*	0	0	1	2.2	0	0	1	0.7
*A. terreus*	1	2	3	6.7	0	0	4	3.1
*A. ustus*	0	0	0	0	3	8.8	3	2.3
*Aspergillus * species	4	7.8	3	6.7	1	2.9	8	6.1
*Chaetomium* species	0	0	1	2.2	0	0	1	0.7
*C. indicum*	0	0	2	4.4	0	0	2	1.5
*C keratinophilum*	5	9.8	7	15.5	5	14.7	17	13.1
*C.* *tropicum*	7	13.7	10	22.2	7	20.6	24	18.5
*Chrysosporium* species	4	7.8	5	11.1	6	17.6	15	11.5
*F. chlamydosporum*	1	1.9	0	0	3	8.8	4	3.1
*F. oxysporum*	0	0	0	0	1	2.9	1	0.7
*F. solani*	2	3.9	0	0	0	0	2	1.5
*Fusarium* species	3	5.9	0	0	0	0	3	2.3
*Paecilomyces* species	0	0	0	0	1	2.9	1	0.7
*P. crustosum*	0	0	1	2.2	0	0	1	0.7
*Penicillium* species	4	7.8	4	8.9	0	0	8	6.1
*Phoma* species	0	0	3	6.7	2	5.9	5	3.8
*Scopulariopsis* species	0	0	3	6.7	0	0	3	2.3
Total	51	100	45	100	34	100	130	100

**Table 2:** Frequency of keratinophilic fungi isolated from Greater Tunb soil based on soil pH

**Species**	**pH**							
	**6-7**		**7.01-8**		**8.01-9**		**>9**	
	**n**	**%**	**n**	**%**	**n**	**%**	**n**	**%**
*Aspergillus *species	0	0	3	20	10	27.8	0	0
*Acremonium* species	0	0	0	0	2	5.5	0	0
*Alternaria* species	0	0	3	20	3	8.3	0	0
*Chrysosporium* species	0	0	6	40	10	27.8	0	0
*Fusarium* species	0	0	2	13.3	4	11.1	0	0
*Aphanoascus* species	0	0	0	0	4	11.1	0	0
*Penicillium* species	0	0	1	6.7	3	8.3	0	0
Total	0	0	15	100	36	100	0	0

**Table 3:** Frequency of keratinophilic fungi isolated from Abu Musa soil based on soil pH

	**pH**									
**Species**	**6-7**			**7.01-8**		**8.01-9**		**>9**		
	**n**	**%**		**n**	**%**	**n**	**%**	**n**		**%**
*Aspergillus* species	0		0	2	15.4	5	15.6		0	0	
*Acremonium* species	0		0	0	0	2	6.2		0	0	
*Phoma* species	0		0	1	7.7	2	6.2		0	0	
*Scopulariopsis* species	0		0	0	0	3	9.4		0	0	
*Chrysosporium* species	0		0	9	69.2	15	46.9		0	0	
*Penicillium* species	0		0	1	7.7	4	12.5		0	0	
*Chaetomium* species	0		0	0	0	1	3.1		0	0	
Total	0		0	13	100	32	100		0	0	

**Table 4:** Frequency of keratinophilic fungi isolated from Sirri soil based on soil pH

**Species**	**pH**							
	**6-7**		**7.01-8**		**8.01-9**		**>9**	
	**n**	**%**	**n**	**%**	**n**	**%**	**n**	**%**
*Aspergillus* species	0	0	3	23	4	19	0	0
*Alternaria* species	0	0	0	0	2	9.5	0	0
*Chrysosporium* species	0	0	6	46	12	57.1	0	0
*Fusarium* species	0	0	3	23	1	4.8	0	0
*Paecilomyces* species	0	0	0	0	1	4.8	0	0
*Phoma* species	0	0	1	7.7	1	4.8	0	0
Total	0	0	13	100	21	100	0	0

## Discussion

Keratinophilic fungi play an important role in the degradation of keratinized residues in the soil. Some types of these fungi can be transmitted to humans as well as animals and cause fungal infections [17]. Up to now, several investigations have been performed in various parts of Iran and other countries indicating the presence of a rich variety of keratinophilic fungal flora in the soils of the studied areas [12-17]. 

Similarly, the present study revealed the presence of keratinophilic fungi in the soil of the investigated islands. Out of the 130 recovered fungal isolates, *Chrysosporium* species had the highest frequency (44.6%). Members of *Chrysosporium* genus are common soil saprobes, many of which are keratino-philic fungi involved in the breakdown of keratinous substrates [24]. 

The frequent occurrence of *Chrysosporium* as a geophilic keratinophilic fungus in this study is in agreement those recorded in the studies examining the soil keratinophilic fungi in several other countries [25-27]. *Chrysosporium* species have a thermotolerant, mesophilic, and hydrophilic nature that could explain the high prevalence of these fungi in the areas with hot and humid climate [28]. 

Mathison and Pugh found that the high distribution of *Chrysosporium* species in coastal soils was due to its enrichment by the molted feathers of birds and fish debris [29]. The high prevalence of *Chrysosporium* species in the soils with neutral or alkaline pH hasalso reported in other studies [29, 30]. Therefore, considering the hot and humid climate and weak alkaline pH of the soil in the islands investigated in the present study, the high prevalence of *Chrysosporium* species in the soil of these regions is justifiable. 

Our study showed that *C. tropicum* (18.5%) was the most prevalent species of *Chrysosporium *in a total of 60 collected soil samples. This fungus has a strong keratinolytic activity and can destroy both cuticle and cortex of the hair [6]. The capacity of *C. tropicum* to utilize keratin has been demonstrated by Agarwal and Deshmukh [31]. This cosmopolitan species has been reported as the most frequent fungus isolated from the soil in several of the previous studies [26, 32, 33]. 

The second most common species of *Chrysosporium* was *C. keratinophilum* (13.1%). The occurrence of *C. keratinophilum* is considered noteworthy due to its tolerance to a wide range of temperatures. This species is usually detected from the soil samples nearby chickens and ducks [34]. This species is generally isolated from human onychomycosis associated with the mycotic superficial invasion of keratinized tissueof the toenail plate [35]. Shadzi *et al.* isolated *C. keratinophilum* (54.2%) as the most frequent keratinophilic fungus from elementary schools and public parks in Isfahan, Iran [36]. 

In another study conducted in Iran by Kachuei *et al.*, *C.keratinophilum* (31.4%) was reported to be the most frequent keratinophilic fungus, followed by *Aspergillus* species [15]. Soomro and Agu reported *Aspergillus* species as the most frequent soil keratinophilic fungi [37, 38]. *A. flavus* (6.1%) had the highest frequency among *Aspergillus* species. This species was the second frequent fungus in the soil of Gorgan, (19.5%) and Gonbad-e Kavus (19%), Iran [39]. *A. flavus* is the common reason for sinusitis in Iran and has the ability to produce mycotoxin [40]. It is also a strong producer of extracellular keratinases in medium with a porcine nail as a source of nitrogen and carbon [41].

In the current study, molecular methods were utilized for the species identification of unknown fungal colony numbers. For example, the application of these methods facilitated the detection of a fungus of *Aphanoascus verrucosus *in the soil of Greater Tunb Island. This keratinophilic fungus has ascospores with anoval shape and a strong and wart wall the pence of which has been reported in the soil of around the world [6]. Cano *et al.* showed that *A. verrucosus* invades the hair through cuticle without the presence of specialized erosive organs and has keratinophilic activity [42]. 

In the present study, none of the dermatophytes were separated from the soil of the studied Islands. This may be attributed to the impact of the environmental factors, such as pH and the organic matter contents, as suggested by many researchers. In our previous study conducted on military forces of the studied Islands, only one case of dermatophytosis was detected, and superficial mycosis were mostly reported [43-45]. Likewise, in a study performed by Soomro and Zaki on the soil keratinophilic fungi in Egypt and Pakistan, no dermatophytes elements were isolated [37, 46]. 

In the present study, we also investigated the relationship between keratinophilic fungi frequency and soil pH. For the first time, Ziegler and Bohme examined the effect of soil pH on keratinophilic fungi and reported that keratinophilic fungi were not observed in the soils with low pH [47]. In line with the previous studies, in the present study, all of the keratinophilic fungi isolated from the soils with a weak alkaline pH were within the range of 7-9. Garg reported that soils with pH of 5.9 are free of the keratinophilic fungi [48]. Kachuei *et al.* isolated keratinophilic fungi from pH of 6-9 [15]. Pakshir *et al.* also, isolated most of the keratinophilic fungi from the soil with the pH range of 7-9 [17].

## Conclusion

As the findings of the present study indicated, the keratinophilic fungal flora of the studied areas was somewhat different from those reported in other parts of Iran. This may be attributed to the climatic and environmental conditions, such as the soil type, substrate, and organic materials in the soil vegetation, as well as fauna and human habitations. The results of this study contributed towards a better concept-tualization of the incidence pattern of keratinophilic fungi in the regions of Iran. Given that no study has investigated this issue, the findings of the present study can be beneficial for the management of public health surveillance, physicians, and epidemiologists.
